# The performance of the alarmin HMGB1 in pediatric diseases: From lab to clinic

**DOI:** 10.1002/iid3.370

**Published:** 2020-11-02

**Authors:** Bo Li, Xin Peng, He Li, Fei Chen, Yuxia Chen, Yingqian Zhang, Kai Le

**Affiliations:** ^1^ Department of Cardiology Children's Hospital of Hebei Province Affiliated to Hebei Medical University Shijiazhuang Hebei China; ^2^ Department of Otolaryngology The Affiliated Children's Hospital of Nanchang University Nanchang Jiangxi China; ^3^ Department of Urology Surgery Qilu Children's Hospital of Shandong University Jinan Shandong China; ^4^ Department of Child Health Care Qilu Children's Hospital of Shandong University Jinan Shandong China; ^5^ Ministry of Education Key Laboratory of Child Development and Disorders, and Chongqing Key Laboratory of Translational Medical Research in Cognitive Development and Learning and Memory Disorders, and Rehabilitation Centre, Children's Hospital Chongqing Medical University Chongqing Yuzhong China; ^6^ Department of Rehabilitation Medicine The First Affiliated Hospital of Nanchang University Nanchang Jiangxi China

**Keywords:** clinic, HMGB1, inflammation, laboratory, pediatric

## Abstract

**Introduction:**

The ubiquitously expressed nonhistone nuclear protein high‐mobility group box protein 1 (HMGB1) has different functions related to posttranslational modifications and cellular localization. In the nucleus, HMGB1 modulates gene transcription, replication and DNA repair as well as determines chromosomal architecture. When the post‐transcriptional modified HMGB1 is released into the extracellular space, it triggers several physiological and pathological responses and initiates innate immunity through interacting with its reciprocal receptors (i.e., TLR4/2 and RAGE). The effect of HMGB1‐mediated inflammatory activation on different systems has received increasing attention. HMGB1 is now considered to be an alarmin and participates in multiple inflammation‐related diseases. In addition, HMGB1 also affects the occurrence and progression of tumors. However, most studies involving HMGB1 have been focused on adults or mature animals. Due to differences in disease characteristics between children and adults, it is necessary to clarify the role of HMGB1 in pediatric diseases.

**Methods and Results:**

Through systematic database retrieval, this review aimed to first elaborate the characteristics of HMGB1 under physiological and pathological conditions and then discuss the clinical significance of HMGB1 in the pediatric diseases according to different systems.

**Conclusions:**

HMGB1 plays an important role in a variety of pediatric diseases and may be used as a diagnostic biomarker and therapeutic target for new strategies for the prevention and treatment of pediatric diseases.

## 1 INTRODUCTION

The high mobility group (HMG) family was first isolated and identified by Ernest Johns, Graham Goodwin, and Clive Sanders in 1973.^1^ The highly conserved high mobility group box 1 (HMGB1) protein with 215 amino acid (aa) residues consisting of two proximal homologous DNA binding domains, A‐box (9–79 aa) and B‐box (95–163 aa), and an uncharged C‐terminal acidic tail (186–215 aa) containing aspartic acid and glutamic acid amino residues was expressed the highest among all HMG family members.^2^ HMGB1 performs a variety of functions that are associated with its redox state, cellular distribution, posttranslational modification, and the type of cells, tissues, and organs in which it is located.^3^ Although research on HMGB1 has continued for decades and it has been explored and validated in various disease models, multiple pathophysiological processes, and signaling pathways, most of these studies were in adult experimental animals, and clinical investigations were also mainly aimed at adults, while research in immature humans and animals is still scarce. After analyzing the PubMed/Medline database by using the following research terms in combination: [(“newborn” OR “neonatal”) OR (“child” OR “children”) OR “pediatric” OR “adolescent”] AND (“hmgb1” OR “hmg1” OR “hmgb‐1” OR “high mobility group box 1” OR “high mobility group box‐1”), we found that the proportion of studies that were truly relevant to pediatric diseases accounted for less than 3%, suggesting that the research on HMGB1 in the diseases of these populations needs urgent attention. The present review classifies and summarizes the published literature associated with the study of HMGB1 in pediatric diseases based on different systems and aims to elucidate its potential role in pediatric diseases.

## 2 SPATIAL DISTRIBUTION OF HMGB1 IN CELLS

The biofunction of HMGB1 is determined by its spatial distribution in cells. As the positioning of HMGB1 changes, its biofunctional changes will also be significant.

### 2.1 Nuclear HMGB1

HMGB1 is ubiquitously expressed in various mammalian cells, and synthetic HMGB1 uses the nucleus as its "armory." In the nucleus, HMGB1 acts as a molecular chaperone by binding to double‐stranded DNA in a nonspecific manner through the A‐box and B‐box,^4^ which are mainly responsible for stabilizing and maintaining nucleosome structure, regulating gene transcription, firming up chromatin, and participating in DNA recombination. Kang et al.^3^ summarized that nuclear HMGB1 is involved in nucleosome stability and sliding, nucleosome number, genome chromatinization, nuclear catastrophe, nucleosome release, DNA binding and bending, V(D)J recombination, gene transcription, gene replication, DNA repair (including DNA mismatch repair, base excision repair, nucleotide excision repair, double‐strand break repair), telomere and telomerase dynamics, gene transfer and gene delivery. In fact, HMGB1 knockout causes lethal hypoglycemia in newborn mice within 24 h of birth; furthermore, although HMGB1‐deficient cell lines appear normal, glucocorticoid receptor‐activated gene expression is impaired, which suggests that HMGB1 is not necessary for overall chromatin structure but is essential for proper transcriptional regulation of specific transcription factors.^5^

### 2.2 Cytosolic HMGB1

Under physiological conditions, the nuclear‐to‐cytoplasmic HMGB1 ratio is approximately 30:1.^6^ HMGB1 is usually located in the nucleus and transfers from the nucleus to the cytosol, including mitochondria and lysosomes, following various stresses. It is currently believed that cytosolic HMGB1 has several biological functions. Cytosolic HMGB1 regulates autophagy and controls mitochondrial dynamics and morphology; autophagic stimuli induce the transport of HMGB1 to the cytosol, and cytosolic HMGB1 binds to Beclin‐1 to induce autophagy and degrade injured organelles and unused proteins.^7^, ^8^, ^9^ Another potential function of cytosolic HMGB1 is participation in the unconventional secretory pathway, which was discovered based on mass spectrometry‐mediated binding partner analysis.^10^ HMGB1 expression is increased and colocalizes with lysosomal proteins in many types of cancer cells. Among the identified cytosolic HMGB1 binding proteins, 9 are related to protein translocation and secretion; among them, annexin A2, myosin IC isoform a, myosin‐9, and the Ras‐related protein Rab10 are directly involved in unconventional protein secretion, which has been confirmed by an immunoprecipitation experiment. HMGB1 is also an important biosensor of intracellular nucleic acids, and DNA or RNA derived from viruses, bacteria, or damaged cells triggers the innate immune response through HMGB1, which is necessary for the subsequent recognition of specific pattern receptors.^11^

### 2.3 Extracellular HMGB1

Since the discovery of HMGB1 as a proinflammatory cytokine and late mediator of sepsis in 1999,^12^ extracellular HMGB1, which is mainly derived by active secretion from immune cells or passive release from damaged/dead cells, has attracted increasing attention as a damage‐associated molecular pattern (DAMP) that induces inflammation in immune cells. Compared with recombinant HMGB1 proteins from prokaryotic sources, native HMGB1 protein from eukaryotic sources showed similar biological activity in vitro.^13^ Extracellular HMGB1 exhibits multiple activities by interacting with different receptors and participates in various pathophysiological processes,^3^ such as cell migration and differentiation, the inflammatory response, tissue regeneration, angiogenesis, bacterial killing, proliferation, cell death, cellular senescence, microRNA effects, efferocytosis, the effects of neurotransmitters and the regulation of the immune response in immune cells (different types of immune cells have the same or unique immune responses). The activation of the immune response mediated by the interaction of HMGB1 with reciprocal receptors mainly occurs in the sterile inflammatory response, while cell growth, migration, invasion, and metastasis are mainly implicated in tumorigenesis.^14^

## 3 POSTTRANSLATIONAL MODIFICATION OF HMGB1

There are extensive epigenetic modifications to lysine residues after HMGB1 translation, of which reversible acetylation is the most important modification. Reversible acetylation is the first modification discovered and the most important modification at present.^15^ HMGB1 contains two nuclear localization sequences (NLSs), which are recognized by the cytosolic transport complex and two nonclassical NESs. Since the affinity of HMGB1 for DNA depends on the degree of HMGB1 acetylation, acetylation can weaken the binding of HMGB1 to DNA and restrict nucleocytoplasmic translocation of HMGB1, determining the localization of HMGB1 under physiological conditions.^16^ The acetylation of two NLS lysine residues of HMGB1 by PCAF, CBP, and histone acetyltransferase p300, mutating six lysines to glutamines to mimic acetylated lysines or the use of deacetylase inhibitors mediate HMGB1 nucleocytoplasmic translocation and secretion in activated monocytes.^17^ Some other acetylases, such as the GCN5^18^ and Janus kinase (JNK)/signal transducer and activator of transcription 1 (STAT1) signaling pathways,^19^ are also involved in the acetylation of HMGB1; in contrast, the deacetylases SIRT1,^20^ SIRT6,^21^ HDAC1, and HDAC4^22^ have been reported to deacetylate HMGB1, thereby limiting the presence of HMGB1 in the nucleus and inhibiting the inflammatory response. Another common posttranslational modification is phosphorylation. Many phosphokinases, such as PKC, CK‐1, CKII, and Cdk5, phosphorylate HMGB1, and phosphorylation of HMGB1 affects its DNA binding/bending affinity and nucleoplasm distribution and release.^23^, ^24^ Phosphorylation reduces the binding of HMGB1 to the nuclear import protein KAP‐α1, which in turn promotes the cytoplasmic relocation of HMGB1 and its eventual secretion.^24^ In addition to acetylation and phosphorylation, posttranslational modifications of HMGB1 also include methylation, ADP‐ribosylation, glycosylation, and oxidation due to changes in three cysteine residues (C23, C45, and C106).

## 4 HMGB1 RELEASE

There are two ways to release HMGB1 into the extracellular space: active release from immune cells after stimulation and passive release from damaged or necrotic cells. The posttranslational modifications of HMGB1, as well as the mechanisms involved in the two release pathways, are different.

### 4.1 Active release

Immune cells such as macrophages, monocytes, neutrophils, dendritic cells (DCs), and natural killer (NK) cells, fibroblasts, or epithelial cells actively release HMGB1 into the extracellular space after stimulation. Stimuli are generally divided into exogenous microbial products such as endotoxin, lysophosphatidylcholine, CpG DNA, or mycobacterial infection, endogenous host stimuli such as interferon‐α (IFN‐α), tumor necrosis factor‐α (TNF‐α), IFN‐γ, IFN‐β, NO, hydrogen peroxide, hyperglycemia, peroxynitrite, hyperlipidemia, kynurenic acid, ATP or neuropeptide Y, and other stimuli such as ethanol, photodynamic therapy, natural DNA or synthetic oligonucleotides, and ultraviolet light.^25^ Due to the lack of a leader signal sequence, HMGB1 cannot be actively secreted through the classic endoplasmic reticulum–Golgi secretory pathway. Instead, active secretion of HMGB1 is triggered by CRM1‐mediated nuclear export and completed by lysosome‐mediated exocytosis. The following mechanisms may respond to the nucleocytoplasmic translocation and release of extracellular HMGB1: posttranslational modifications, CRM1‐mediated nuclear export (e.g., Hsp72 overexpression suppresses CRM1 translocation and the interaction between HMGB1 and CRM1 in macrophages following lipopolysaccharide [LPS], or TNF‐α treatment^26^), the reactive oxygen species (ROS) signaling pathway (e.g., H_2_O_2_ actives the mitogen‐activated protein kinase [MAPK], and nuclear factor‐κB [NF‐κB] pathways, which in turn promote HMGB1 release in macrophages and monocytes^27^), the calcium signaling pathway (e.g., the inhibition of calcium signaling by inhibitors such as STO609 and CV159 or knockdown/knockout of CaMK I and IV reduce HMGB1 release and protect animals from ischemia/reperfusion injury or sepsis^28^), the nitric oxide (NO) signaling pathway, mechanisms dependent on TNF‐α, Notch, NF‐κB, MAPK, STAT, inflammasome, p53, PPAR or lysosome, and cell‐mediated release of HMGB1 (the interplay between dying cells and immune cells also induces HMGB1 release).

### 4.2 Passive release

In addition to the active secretion of HMGB1 after cells are stimulated, HMGB1 can also be passively released by necrotic or damaged cells.^29^ A number of stimuli that cause cell damage, including physical factors such as ischemia–reperfusion, hypoxia, nonpenetrating trauma, toxemia, sterile tissue impairment, irradiation, chemotherapy, hyperthermia, hyperpressure, glucose deprivation, and foreign matter and chemical factors such as free fatty acids, Bacillus Calmette–Guerin, viral infections, toxins, and ATP, lead to the passive release of HMGB1. The possible mechanisms involved in the passive release are summarized as follows: a PARP1‐dependent mechanism (compared with that of wild‐type cells, the deletion of PARP1 in MEFs or the use of PARP inhibitors significantly inhibits the alkylated DNA damage‐induced translocation and release of HMGB1), an RIP3‐dependent mechanism (related to programmed necrosis), a cathepsin‐dependent mechanism, an antioxidant enzyme‐dependent mechanism (ROS not only induce HMGB1 secretion but also promote the release of HMGB1 during cell death such as necrosis, necroptosis, and apoptosis.), a DNase‐dependent mechanism, a caspase‐dependent mechanism (related to pyroptosis), and an ATG‐dependent mechanism (related to autophagy).

## 5 ROLE OF HMGB1 IN PEDIATRIC DISEASES

Currently, HMGB1 expression has been shown to be very important in the genesis and promotion of different inflammatory diseases, including different types of pediatric diseases. The role of HMGB1 in the initiation and development of multiple pediatric diseases has been reported by different research groups worldwide. The role of HMGB1 in pediatric diseases has been described in detail below based on the different systems (summarized in Table 1).

**Table 1:** HMGB1 performance in pediatric disease

System or disease type	Disease	Changes in HMGB1 levels in human specimens	Supporting literature	Perspectives
Infectious diseases	HFMD	Serum ↑	^[31]^	Assist in determining the severity and prognosis of the disease
	HSV infection	Serum ↑	^[33]^	Assist in determining the severity and prognosis of the disease
	RSV infection	Nasopharyngeal aspirates ↑	^[34]^	Therapeutic targeting of HMGB1 pathway
	Falciparum malaria	Serum ↑	[^39^, ^40^, ^41^]	Assist in determining the severity and prognosis of the disease
	Sepsis	Serum ↑	[^42^, ^45^, ^46^, ^47^]	Late‐stage prognostic factor or as a marker of therapeutic response
Digestive system	NEC	Ileum tissue ↑	^[50]^	Marker of severity and prognosis and a target for early intervention
		Serum ↑	[^52^, ^53^]	
	IBD	Feces ↑	[^56^, ^57^]	Assist in determining the severity of the disease and sensitivity to treatment
	CD	Serum ↑	^[59]^	Fecal HMGB1 may play a supplemental role of serological testing in the management of CD children
		Feces ↑	^[62]^	
	AA	Serum ↑	[^64^, ^65^]	Assist in determining the severity and biomarker for the diagnosis of disease, especially for patients with normal WBC
	BA	Serum and liver biopsy tissues ↑	^[71]^	Therapeutic targeting of HMGB1 pathway and as a new diagnostic marker for BA
	NAFLD	Blood ↑	^[75]^	Controversial
		Serum →	^[76]^	
Respiratory system	Pneumonia	Peripheral blood ↑	^[79]^	Diagnostic marker for refractory *M. pneumoniae* pneumonia
		Serum (severe pneumonia among children infected with H1N1 influenza virus) ↑	^[80]^	
	Bronchiolitis	Nasal secretions ↑	^[81]^	Assist in grading the severity of acute bronchiolitis
	BPD	Endotracheal aspiration ↑	^[86]^	HMGB1 inhibition as therapy
	Asthma	Sputum ↑	[^89^, ^90^]	Double‐edged sword
	NRDS	Serum ↑	^[96]^	Biomarker for diagnosis, evaluation, and prognosis of NRDS
Nervous system	FS	Blood ↑	[^101^, ^102^, ^103^]	Assist in determining the severity and prognosis of the disease
	Epilepsy	Serum ↑	[^103^, ^106^]	Assist in determining the severity and prognosis of the disease
	TBI	Cerebrospinal fluid ↑	^[114]^	As a prognostic factor
	ASD	Serum ↑	[^120^, ^121^, ^123^]	Biomarker of the severity of ASD symptoms
	Meningitis	Cerebrospinal fluid ↑	[^126^, ^127^]	Assist in determining the severity of the disease and as a marker of therapeutic response
	PA	Serum ↑	^[131]^	Assist in determining the severity of the disease and as a marker of therapeutic response
	HIE	Umbilical cord blood ↑	^[133]^	Therapeutic targeting of HMGB1 pathway
	Premature brain damage	Umbilical cord blood ↑	^[138]^	Therapeutic targeting of HMGB1 pathway
Autoimmune disorders and vasculitis	JIA	Serum and synovial fluid ↑	[^140^, ^141^]	Assist in determining the severity of the disease and as a marker of therapeutic response
		Serum ↑	^[142]^	
	SLE	Serum ↑	[^140^, ^145^, ^146^]	Assist in determining the severity of the disease and as a marker of therapeutic response
	KD	Serum ↑	[^150^, ^151^]	Markers for the revealing of IVIG resistance in advance
	MAS	Serum (ds‐HMGB1) ↑	^[155]^	Therapeutic targeting of HMGB1 pathway
	HSP	Serum ↑	[^157^, ^158^]	Therapeutic targeting of HMGB1 pathway
	AR	Nasal lavage fluid ↑	^[160]^	Therapeutic targeting of HMGB1 pathway
	VKC	Serum and lacrimal fluid ↑	[^162^, ^163^]	Therapeutic targeting of HMGB1 pathway
Endocrine system	MS	Blood ↑	^[165]^	Diagnostic marker
Cancer	Osteosarcoma	Osteosarcoma tissues ↑	^[172]^	HMGB1 plays an important role in the occurrence, progression, and sensitivity of osteosarcoma to chemotherapy
	Retinoblastoma	Retinoblastoma tissues ↑	^[178]^	As a factor of chemotherapeutic resistance
	Neuroblastoma	Neuroblastoma tissues ↑	^[182]^	Therapeutic targeting of HMGB1 pathway
	Leukemia	Serum ↑	[^185^, ^186^, ^187^]	Therapeutic targeting of HMGB1 pathway
	NHL	Lymph node tissues ↑	^[190]^	Therapeutic targeting of HMGB1 pathway
Skin disease	ETN	Skin lesions (nucleocytoplasmic relocation in keratinocytes and macrophages)	^[193]^	Therapeutic targeting of HMGB1 pathway
	AEDS	Serum ↑	^[195]^	Assist in determining the severity of the disease and as a marker of therapeutic response
	EB	Serum ↑	^[197]^	Assist in determining the severity of the disease and as a marker of therapeutic response
Others	SCD	Plasma ↑	^[203]^	Assist in determining the severity of the disease
	Peritonitis	Serum and peritoneal fluid ↑	^[205]^	Assist in diagnosis of the disease and as a marker of therapeutic response
	Alcohol abuse	Human post‐mortem orbitofrontal cortex ↑	[^206^, ^207^]	Therapeutic targeting of HMGB1 pathway

Abbreviations: AA, acute appendicitis; AEDS, atopic eczema/dermatitis syndrome; AR, allergic rhinitis; ASD, autism spectrum disorder; BA, biliary atresia; BPD, bronchopulmonary dysplasia; CD, celiac disease; EB, epidermolysis bullosa; ETN, erythema toxicum neonatorum; FS, febrile seizures; HFMD, hand‐foot‐and‐mouth disease; HIE, hypoxic–ischemic encephalopathy; HMGB1, high‐mobility group box protein 1; HSP, Henoch–Schönlein purpura; HSV, Herpes simplex virus; IBD, inflammatory bowel disease; JIA, juvenile idiopathic arthritis; KD, Kawasaki disease; MAS, macrophage activation syndrome; MS, metabolic syndrome; NAFLD, nonalcoholic fatty liver disease; NEC, neonatal necrotizing enterocolitis; NHL, non‐Hodgkin's lymphoma; NRDS, neonatal respiratory distress syndrome; PA, perinatal asphyxia; RSV, respiratory syncytial virus; SCD, sickle cell disease; SLE, systemic lupus erythematosus; TBI, traumatic brain injury; VKC, vernal keratoconjunctivitis; ↑, upregulation; →, unchanged.

### 5.1 HMGB1 and infectious diseases

#### 5.1.1 Hand–foot‐and‐mouth disease

Hand–foot‐and‐mouth disease (HFMD) is a common pediatric disease caused by enterovirus infections and mainly occurs in the Asia‐Pacific region dominated by China. Severe cases are usually accompanied by several neurological complications (meningitis, encephalitis, and neurogenic pulmonary edema) and circulatory disorders and occasionally lead to death.^30^ Zheng et al.^31^ reported that serum HMGB1, IL‐6, and TNF‐α in children with HFMD caused by EV71 infection were significantly increased, which was associated with the severity of the disease, suggesting that HMGB1 may be involved in the inflammatory mechanism of HFMD induced by EV71, and its level could be used as a clinical indicator to determine the severity and prognosis of HFMD.

#### 5.1.2 Herpes simplex virus infection

Herpes simplex virus (HSV) often affects premature neonates and full‐term neonates. There are three types of HSV infection: lesions limited to the skin, eye, and mouth mucosa, central nervous system (CNS) infection, and systemic disseminated infection.^32^ The serum level of HMGB1 in neonates with systemic disseminated HSV infection increased progressively over time, and the upregulation of HMGB1 preceded that of cytochrome C.^33^

#### 5.1.3 Respiratory syncytial virus infection

Respiratory syncytial virus (RSV) is a causative agent that induces severe respiratory infections in infants, elderly individuals and immunocompromised people worldwide. The pulmonary inflammatory immune response plays an important role in the outcome of RSV infection. It has been clinically shown that the level of HMGB1 in nasopharyngeal aspirates of children with bronchiolitis caused by RSV was significantly higher than that of patients without lower respiratory tract infection and was correlated with clinical severity.^34^ RSV strongly induces HMGB1 expression both in vivo and in vitro, and the inhibition of HMGB1 blocks the upregulation of HMGB1 in immortalized or primary human bronchial epithelial cells infected with RSV; this was associated with reduced viral replication.^34^, ^35^ In addition, it was also shown that RSV‐infected human airway epithelial cells release HMGB1 in a paracrine manner to activate immune cells to secrete inflammatory mediators to promote the inflammatory cascade that depends on TLR4/NF‐κB signaling pathway activation,^36^, ^37^ which may provide a new strategy for the prevention of the RSV‐induced inflammatory response at the molecular level.

#### 5.1.4 Falciparum malaria

Falciparum malaria, caused by *Plasmodium falciparum* infection, remains the leading cause of morbidity and mortality from malaria globally, and persistent, intense and complicated inflammation is associated with harmful clinical outcomes.^38^ As an inflammatory factor, HMGB1 was shown to be elevated in the serum of African children with *falciparum* malaria and was associated with severe complications, while HMGB1 levels gradually decreased during the recovery period.^39^, ^40^, ^41^ In vitro experiments showed that *Plasmodium*‐parasitized erythrocytes could induce human peripheral monocytes to release HMGB1, demonstrating that the increase in HMGB1 in patients with persistent *P. falciparum* infection may prolong the inflammation and fever of malaria, but unfortunately, treatment with the HMGB1 neutralizing antibody in an experimental mouse model of severe malaria did not reduce mortality.^39^ The role of HMGB1 in *falciparum* malaria remains to be further explored.

#### 5.1.5 Sepsis

Since the discovery of HMGB1 as a late‐stage inflammatory mediator of sepsis, the role of HMGB1 in childhood sepsis has also received considerable attention. Carrol et al.^42^ attempted to explore the use of HMGB1 as a marker for the diagnosis of children with severe bacterial infection in Latvia. However, the researchers did not obtain positive results because HMGB1 was not better than procalcitonin in identifying severe sepsis. Pavare et al.^43^ also showed that LPS binding protein, IL‐6, and CRP are associated with the severity of infection in children, but HMGB1 does not seem to be important. The possible reason is that the researchers only analyzed early inflammation, while HMGB1 is often elevated in the late stage of sepsis, lasts for a long time, and can mediate sepsis‐associated anemia by interfering with erythropoiesis.^44^ In contrast, Zhuo et al.^45^ reported that HMGB1 in peripheral monocytes in neonatal sepsis increased and activated the TLR4/NF‐κB pathway to produce inflammatory factors, while simvastatin combined with human‐derived kallikrein binding protein was used to treat children with burns complicated with sepsis and reduced the abnormally elevated HMGB1 in endothelial cells.^46^ In fact, HMGB1 is secreted by endotoxin‐activated macrophages, and secretion increases 20–72 h after infection.^47^ Edaravone's ability to maintain average arterial pressure and prolong the survival time of neonatal mice with sepsis caused by cecal ligation and perforation was partly attributed to its prevention of HMGB1 elevation.^48^ Therefore, HMGB1 should be evaluated as a late‐stage prognostic factor or as a marker of the therapeutic response, rather than an early marker of sepsis. In general, sepsis has been studied in animal models and adults, while there are limited data on the pediatric population; thus, more research is needed to determine the role of HMGB1 in sepsis.

### 5.2 HMGB1 and diseases of the digestive system

#### 5.2.1 Neonatal necrotizing enterocolitis

Neonatal necrotizing enterocolitis (NEC) is the leading cause of death and disability from gastrointestinal disease in premature infants, and approximately 10% of premature infants develop NEC, severe cases of which lead to death. Although the pathogenesis of NEC has not been fully elucidated, it is currently believed that many inflammatory mediators are involved, such as fecal calmodulin, IL‐6, TNF‐α, or transforming growth factor‐β.^49^ As an important inflammatory mediator, HMGB1 is closely related to the occurrence, development, and complications of NEC. The level of ileal HMGB1 in NEC infants was elevated, and the level of HMGB1 and RAGE in the distal ileum of neonatal rats with NEC was also upregulated, while inhibiting HMGB1 by semapimod partially protected intestinal epithelial cells from death.^50^ A study by Downard et al.,^51^ suggested that the messenger RNA (mRNA) levels of HMGB1 and TLR4 in the distal ileum were increased in NEC. HMGB1 in the peripheral blood was also abnormally elevated in NEC, and premature infants with NEC might develop more serious cognitive impairment and lung injury, both of which are mediated by HMGB1 released by intestinal injury through the activation of TLR4.^52^, ^53^ Additionally, the administration of the HMGB1 inhibitor glycyrrhizin inhibited the TLR4/NF‐κB signaling pathway and thereby inhibited NLRP3, ultimately alleviating intestinal inflammation in NEC.^54^ HMGB1 has the potential to be a marker of NEC severity and prognosis, as well as a target for early intervention.

#### 5.2.2 Inflammatory bowel disease

Inflammatory bowel disease (IBD), which includes ulcerative colitis (UC), and Crohn's disease, is a group of chronic inflammatory disorders of the gastrointestinal tract that begin most often during adolescence and young adulthood.^55^ The incidence of IBD has increased dramatically in the past 50 years, with a gradual decline in median age. IBD symptoms in intestinal and extraintestinal organs have significant impacts on the growth, development, bone health, and psychosocial function of children. Current measurement of inflammation in IBD usually requires invasive surgery, such as ileal colonoscopy, which often causes discomfort and inconvenience. The importance of judging the severity of IBD through the detection of inflammatory markers in feces is receiving increasing attention. Vitali et al.^56^ revealed that fecal HMGB1 was significantly increased in children with Crohn's disease and UC but was not detected in healthy controls, and fecal HMGB1 was related to calprotectin levels. Although no changes were found in the mRNA and protein levels of HMGB1 in inflamed biopsy tissue, the level of cytoplasmic HMGB1 was significantly increased, suggesting that HMGB1 in feces was actively secreted by intestinal cells rather than generated by de novo synthesis.^56^ Later, Palone et al.^57^ also discovered a similar phenomenon in the feces of children and adults with Crohn's disease and UC, and the HMGB1 levels were closely related to the severity of disease. Furthermore, in patients with clinical and endoscopic remission, only fecal HMGB1 levels showed a strong correlation with the histological score of the degree of inflammation.^57^ These findings suggest that fecal HMGB1 may be a reliable biomarker for IBD‐associated intestinal inflammation and indicate histological changes in patients with clinical and endoscopic remission.

#### 5.2.3 Celiac disease

Celiac disease (CD) is a gluten‐induced bowel disease with a prevalence of 1% in children that mainly causes chronic inflammation of the small intestinal mucosa and leads to abdominal pain, chronic diarrhea, anemia, malnutrition, and parenteral symptoms.^58^ Lifelong adherence to a gluten‐free diet (GFD) is the key treatment for CD patients, both to promote mucosal healing and prevent complications. The serum HMGB1 level in children with CD is significantly higher than that in healthy children, and the serum HMGB1 in children with typical clinical symptoms is also significantly different from that in children with atypical clinical symptoms; additionally, HMGB1 reflects the histological severity of disease according to the Marsh classification.^59^ Since CD often leads to a lower response to hepatitis B virus (HBV) than that of healthy individuals,^60^ Manti et al.^61^ found a significant correlation between serum HMGB1 level and HBV vaccination responsiveness, and nonresponders had significantly higher serum HMGB1 levels than responders, indicating that HMGB1 might represent a new marker that reflects immune function impairment resulting in HBV vaccination failure. In addition, Palone et al.^62^ revealed that fecal HMGB1 levels in children with CD were significantly increased and strongly correlated with serum anti‐tTGA levels. During the 12‐month follow‐up period after GFD treatment, HMGB1 could no longer be detected in the feces of 75% of CD children, while the remaining 25% of children had decreased HMGB1 levels; half of them reported gastrointestinal symptoms such as bloating, upper abdominal pain, and constipation but their serum anti‐tTGA levels were normal,^62^ further supporting that fecal HMGB1 may play a supplemental role in serological testing in the management of CD children.

#### 5.2.4 Acute appendicitis

Acute appendicitis (AA) is a common surgical emergency in children. The characteristics of AA in children frequently lead to delayed diagnosis and a high rate of misdiagnosis. At present, some routine laboratory tests, such as white blood cell (WBC) counts, polymorphocyte counts, and CRP, are nonspecific, and whether diagnostic imaging tests reduce complications is still controversial^63^; therefore, researchers are trying to find new serum biomarkers for the early diagnosis of AA. Hu et al.^64^ discovered that serum HMGB1 levels in children with AA were significantly elevated, and for the diagnosis of AA, the sensitivity and specificity of serum HMGB1 were 71.4% and 82.9%, respectively, the best cutoff value was 28.0 ng/ml, and the area under the ROC curve (AUC) was 0.765 (95% confidence interval [CI]: 0.638–0.893) with an accuracy of 77.8%. In addition, the serum HMGB1 level of children with perforated appendicitis was higher than that of those without perforation,^65^ which was consistent with the findings of previous studies in adult AA patients,^66^, ^67^ suggesting that serum HMGB1 may be implicated in the severity of AA and is an important biomarker for the diagnosis of AA, especially for patients with normal WBC counts.

#### 5.2.5 Biliary atresia

In biliary atresia (BA), a common neonatal bile duct disease, pathological changes in fibrosis and catheter system occlusion result in bile flow obstruction and cholestasis, causing progressive hepatobiliary injury, which is the most common predisposing factor for liver transplantation in infants.^68^ Some common viral infections and increased systemic inflammatory responses are currently considered to play key roles in the progression of BA.^69^, ^70^ HMGB1 levels were found to be significantly elevated in serum and liver biopsy tissues of BA infants compared with those of healthy infants, and serum HMGB1 levels were positively correlated with γ‐glutamyltransferase levels.^71^ HMGB1 expression was also increased in rotavirus infection‐induced BA model animals.^72^ Abnormally increased levels of HMGB1 were released from injured bile duct cells and macrophages, activating the HMGB1/TLRs/MAPK signaling pathway to further activate NK cells. Due to the inability of immature NK cells to eliminate bile duct cells infected with rotavirus, persistent rotavirus infection in bile ducts occurred. With increasing age, HMGB1 promoted the maturation of NK cells in mice, leading to increased and persistent immune responses in bile duct cells that eventually induced BA.^72^ These findings demonstrate that HMGB1 plays a key role in the pathogenesis of BA. The correlation between serum HMGB1 levels and γ‐glutamyltransferase levels can be used as a new diagnostic marker for BA.

#### 5.2.6 Nonalcoholic fatty liver disease

Nonalcoholic fatty liver disease (NAFLD), one of the most common chronic liver diseases in the world, may cause cirrhosis, hepatoma or hepatic failure^73^ and affects 3%–12% of children worldwide.^74^ Alisi et al.^75^ found in a cross‐sectional study of 110 children with NAFLD confirmed by biopsy that the blood HMGB1 level in patients was significantly higher than that in controls, and there was an apparent association with a high degree of fibrosis. Paradoxically, the results of a cross‐sectional study conducted by Yates et al.^76^ showed that there was no significant correlation between serum HMGB1 levels and histological characteristics such as steatosis, balloon‐like degeneration, inflammation, fibrosis, or steatohepatitis in children and adults with NAFLD and that serum HMGB1 levels did not change significantly during drug therapy. In the longitudinal analysis, it was also found that the changes in serum HMGB1 level were not related to histological improvements, NASH regression or ALT level. The results of HMGB1 in pediatric NAFLD are controversial, and further research is needed to clarify the role of HMGB1 in NAFLD.

### 5.3 HMGB1 and diseases of the respiratory system

#### 5.3.1 Pneumonia/bronchiolitis

In developing countries, pneumonia remains the most important cause of death among children under 5 years of age,^77^ and early identification and treatment of pneumonia patients is crucial to decreasing mortality. Zhou et al.^78^ found that HMGB1 distinguishes children with bronchial pneumonia with coinfection and single infection in a study of the accuracy and effectiveness of 13 markers to distinguish viral and bacterial pneumonia in Han children. Peripheral blood HMGB1 levels in children with refractory *Mycoplasma pneumoniae* pneumonia were increased; the AUC for HMGB1 in the diagnosis of refractory *M. pneumoniae* pneumonia was 0.876, with a sensitivity of 0.833 and specificity of 0.824.^79^ The results of in vitro experiments indicated that the HMGB1/TLR2 pathway may be involved in the occurrence of refractory *M. pneumoniae* pneumonia, indicating that HMGB1 was a good diagnostic biomarker for distinguishing refractory *M. pneumoniae* pneumonia from nonrefractory *M. pneumoniae* pneumonia.^79^ In addition to clinical studies, HMGB1 levels in bronchoalveolar lavage fluid and lung tissue were significantly increased in animal models of pneumonia induced by adenovirus infection, and the expression of the receptors TLR4/9 and RAGE also varied with the changes in HMGB1, confirming that the HMGB1 signaling pathway plays a role in the occurrence and development of adenovirus‐mediated pneumonia.^80^ The level of HMGB1 in the nasal secretions of children with bronchiolitis requiring hospitalization was significantly higher than those of children who were allowed to leave the hospital after emergency treatment, which makes it possible to grade the severity of acute bronchiolitis in children by measuring HMGB1 in nasal secretions.^81^ Furthermore, HMGB1 was significantly increased in the serum of children with severe pneumonia who were infected with H1N1 influenza virus and was significantly associated with the upregulation of 10 other cytokines.^80^ These findings suggest that HMGB1 plays an important role in the pathogenesis of pneumonia, especially refractory pneumonia or severe pneumonia and bronchiolitis.

#### 5.3.2 Bronchopulmonary dysplasia

Bronchopulmonary dysplasia (BPD) is a major cause of chronic lung disease in neonates, especially premature infants. At present, BPD has become the leading cause of death in premature infants after birth, and its harmful effects last into adulthood.^82^ Various factors, such as hyperoxia or excessive inflammation caused by mechanical ventilation, lead to lung injury characterized by emphysema and interstitial edema and fibrotic lung collapse.^83^ HMGB1 was considered to be a late mediator in an adult animal model of lung injury induced by ventilators and hyperoxia.^84^, ^85^ Similarly, in premature infants requiring mechanical ventilation, HMGB1 levels were significantly lower in endotracheal aspirates from those without BPD than those with BPD, and dexamethasone treatment had no effect on HMGB1 levels.^86^ Premature mice with BPD induced by hyperoxia also confirmed that the protein and mRNA expression of HMGB1 was significantly increased in the lung.^87^ In addition, Yu et al.^88^ also demonstrated that the increase in HMGB1 levels in the whole lung after hyperoxia exposure was related to impaired alveolar development and abnormal elastin production, and inhibiting HMGB1 downregulated the inflammatory response and partially improved alveolar development.

#### 5.3.3 Asthma

As an important inflammatory mediator, the role of HMGB1 in asthma has also received attention; that is, the sputum HMGB1 levels of asthmatic children were significantly elevated compared with those of the healthy control group^89^, ^90^ and were positively correlated with the total IgE level,^90^ and the sputum HMGB1 levels of children with mild, moderate, or severe symptoms were strictly negatively correlated with the lung function index.^90^ Inhaled glucocorticoid therapy could reduce sputum HMGB1 levels in moderate to severe asthma.^89^ Additionally, animal models of asthma showed that ovalbumin induced the expression of HMGB1, TLR2, and TLR4 in the lung, while inhibiting HMGB1 reduced the number of Th1, Th2, and Th17 cells to dampen airway inflammation.^91^ Vitamin D mitigated airway inflammation and lung tissue apoptosis in asthmatic mice by inhibiting the activation of the HMGB1/TLR4/NF‐κB pathway.^92^ Although the role of HMGB1 in asthma‐associated inflammation has gradually been revealed, it cannot be ignored that Ojo et al.^93^ claimed that although airway exposure to irritants or allergens may compress or damage cells in the airway and cause the release of HMGB1, the HMGB1 signaling axis involves TLR4 and RAGE and the coordinated induction of extracellular signal‐regulated protein kinases 1 and 2 (ERK1/2) and JNK that underpins epithelial wound closure through a mechanism that involves the loss of epithelial cell–cell contact and the induction of the synthesis of ECM proteins (fibronectin and laminin‐5) and ECM receptors, and epithelial repair is essential for reconstructing and restoring the integrity of the airway epithelium after injury, which would otherwise contribute to the development of obstructive airway disease. These findings indicate that HMGB1 is a double‐edged sword in the pathogenesis of asthma, and its specific mechanism still needs further elucidation.

#### 5.3.4 Neonatal respiratory distress syndrome

A variety of inflammatory mediators and cytokines are thought to play important roles in the pathophysiology of neonatal respiratory distress syndrome (NRDS)^94^, ^95^; however, these early inflammatory mediators appear early and are present for a short time, which only contributes to the early diagnosis of NRDS, limiting the evaluation of the severity and prognosis of NRDS. HMGB1, which is a late inflammatory mediator, was considered to be an indicator of NRDS severity and prognosis. The serum HMGB1 levels of NRDS infants were significantly higher than those of the controls and were associated with disease severity.^96^ The serum HMGB1 levels of NRDS infants who died were significantly higher than those of NRDS survivors.^96^ Receiver operating characteristic (ROC) analysis showed that the AUC value for predicting NRDS was 0.846 (95% CI: 0.755–0.936), and the best cutoff value for serum HMGB1 to predict NRDS was 625.3 pg/ml; the AUC value used to predict the risk of death in NRDS children was 0.916 (95% CI: 0.813–1.000), and the best cutoff value for serum HMGB1 to predict death was 772.2 pg/ml.^96^ Another similar study reported consistent results,^97^ which suggested that serum HMGB1 levels could predict the occurrence and death of NRDS and had the potential to be a biomarker for the diagnosis, evaluation, and prognosis of NRDS.

### 5.4 HMGB1 and diseases of the nervous system

#### 5.4.1 Febrile seizures and epilepsy

Although long granted the status of immune privilege, the CNS is not absolutely isolated from the immune system. A growing body of evidence suggests that this system is sensitive to seizures and epilepsy and that the molecular mediators of inflammation and immunity can be used as appropriate targets for the development of new antiepileptic drugs that are efficacious and potentially disease modifying. Febrile seizures (FS) is the most common form of convulsive seizures in childhood, with an incidence of approximately 2%–5% in children under 6 years of age.^98^ The complex interactions between immune‐inflammatory processes, cytokine activation, and genetic factors play important roles in the development of FS.^99^, ^100^ The levels of blood HMGB1 and IL‐1β in children with FS after 30 min of seizure attacks were significantly higher than those with fever only, and these two inflammatory indicators were correlated with each other.^101^, ^102^, ^103^ HMGB1 contributes to the onset of FS and plays an important role in the occurrence of secondary epilepsy associated with the prolongation of FS,^104^ and the occurrence of adult epilepsy is related to the recurrence of FS in childhood.^105^ Similar to that of FS, serum HMGB1 was significantly elevated within 24 h of seizures in children with epilepsy and had the potential to predict seizure frequency and prognosis more accurately than IL‐1β.^103^, ^106^ Data from different experimental models of acute and chronic epilepsy have shown that HMGB1‐TLR4 signaling in the hippocampus plays a key role in the generation and recurrence of seizures.^107^ The results of analyses of surgically obtained hippocampal tissue from children with medial temporal lobe epilepsy (MTLE) and experimental animals indicated that HMGB1, TLR4, p‐p38MAPK, and inflammatory factors were increased, suggesting that the HMGB1/TLR4/p38MAP signaling pathway plays an important role in the pathogenesis of MTLE.^108^ In addition, in children with refractory epilepsy caused by focal cerebral cortical dysplasia, it was also shown that the level of HMGB1 signaling pathway‐related protein in the lesion tissue was significantly upregulated and was accompanied by increased cytoplasmic localization of HMGB1 in neurons and astrocytes.^109^, ^110^ The acetylated disulfide HMGB1 isoform was continuously expressed in patients with drug‐refractory epilepsy and could be used as a biomarker for human epilepsy occurrence and resistance.^111^

#### 5.4.2 Traumatic brain injury

Traumatic brain injury (TBI) is the leading cause of death and permanent disability in children.^112^ Several markers of neuroinflammation are associated with poor prognosis in children with severe TBI.^113^ The cerebrospinal fluid (CSF) HMGB1 level in children with TBI was increased and was negatively correlated with a favorable outcome on the Glasgow Outcome Scale.^114^ A TBI model in adolescent rats was used to show that HMGB1 nucleocytoplasmic translocation and microglial activation occurred after brain injury.^115^ By inhibiting HMGB1, TBI‐induced glial cell activation and short‐term spatial memory and motor learning defects were ameliorated,^116^ indicating that HMGB1 is a danger signal in TBI.

#### 5.4.3 Autism spectrum disorder

Autism spectrum disorder (ASD) is a common neurodevelopmental disorder whose core symptoms include difficulties in social communication and social interaction and restricted and repetitive behaviors, interests, or activities^117^ and currently has no specific treatment. Immune dysregulation is thought to be involved in the pathophysiological process of ASD.^118^, ^119^ HMGB1 levels are increased in adult and pediatric ASD patients and are independently associated with autism scores that reflect social deficits.^120^, ^121^ Monocyte cultures from children with ASD were more sensitive to signaling through selected TLRs, indicating dysfunction in monocyte pathogen recognition and/or TLR signaling pathways.^122^ Additionally, in ASD children, there was a strong correlation between HMGB1 and the reduction in plasma epithelial growth factor levels, which was associated with the severity of some symptoms.^123^ In addition to core symptoms, higher HMGB1 levels can lead to more severe chronic gastrointestinal dysfunction in children with ASD than those with lower HMGB1 levels.^121^ High serum levels of HMGB1 may be biomarkers of the severity of ASD symptoms.

#### 5.4.4 Meningitis

The excessive inflammatory response caused by bacterial meningitis, including the release of proinflammatory factors and matrix metalloproteinases, is the main cause of brain damage. Inflammatory factors also destroy the blood–brain barrier, which allows peripheral immune factors to infiltrate the CNS, further maintaining the injury caused by the inflammatory response.^124^ Injured cells release DAMPs, which in turn activate immune cells to secrete more inflammatory factors, leading worse damage.^125^ The levels of HMGB1 in the CSF of children with bacterial meningitis or aseptic meningitis were all increased, but the concentrations of HMGB1 in the CSF of individuals with bacterial meningitis was four times that of individuals with aseptic meningitis, and there was a significant correlation between CSF HMGB1, WBC counts, and glucose in bacterial meningitis cases.^126^, ^127^ The level of HMGB1 and other inflammatory mediators in the hippocampus was also significantly increased in young mice with pneumococcal meningitis.^128^

#### 5.4.5 Perinatal asphyxia and hypoxic–ischemic encephalopathy

Severe perinatal asphyxia (PA) causes irreversible impairments in neonatal tissues/organs, leading to multiple organ failure and causing millions of neonatal deaths worldwide each year.^129^ Pathophysiological studies have shown that the damage caused by asphyxia is mainly related to inflammation induced by ischemia–reperfusion injury.^130^ The level of serum HMGB1 in neonates with asphyxia was significantly higher than that in normal delivery neonates without asphyxia.^131^ HMGB1 levels were positively correlated with other indicators, such as TNF‐α and NSE and were higher in newborns with abnormal amplitude integrated electroencephalogram (aEEG) 6 h postpartum than in those with normal aEEG.^132^ Dynamic monitoring of HMGB1 combined with aEEG can provide a basis for the early diagnosis of brain damage caused by neonatal PA. PA is an important cause of neonatal hypoxic–ischemic encephalopath (HIE), and HMGB1 is also significantly increased in the umbilical cord blood in neonatal HIE.^133^ In addition, animal models of HIE and in vitro experiments have shown that neurons, astrocytes, and activated microglia all exhibit nucleocytoplasmic translocation and release of HMGB1,^134^, ^135^ which is inhibited by treatment with hypothermia^133^ or resveratrol,^134^ suggesting that HMGB1 may be a useful indicator of the severity of the disease and treatment efficacy.^134^, ^136^

#### 5.4.6 Premature brain damage

Intrauterine infection and subsequent inflammation is one of the causes of premature brain damage, which mainly involves the release of cytokines and chemokines, as well as the activation of microvascular endothelial cells and leukocytes.^137^ Severe brain injury is accompanied by increased levels of umbilical cord blood HMGB1 and reduced levels of sRAGE. In addition, compared with that of preterm infants without intraventricular hemorrhage (IVH), the concentration of HMGB1 in the umbilical cord blood of premature infants with IVH was significantly increased, suggesting that the increased HMGB1 levels may be related to IVH in preterm infants.^138^

### 5.5 HMGB1, autoimmune disorders, and vasculitis

#### 5.5.1 Juvenile idiopathic arthritis

The role of the immune response in juvenile idiopathic arthritis (JIA) is self‐evident, and research on HMGB1 in JIA has occurred for more time than that in other pediatric diseases.^139^ High levels of HMGB1 were observed in the serum and synovial fluid of children with JIA,^140^, ^141^ and HMGB1 levels were highest in the early stages of the disease, independent of the course of the disease.^141^ There is a positive correlation between serum HMGB1 and ESR, CRP, and α2 globulin, and high levels of serum HMGB1 are also associated with hepatosplenomegaly or serositis in systemic onset type JIA.^140^ A 10‐year follow‐up study of children with JIA suggested that HMGB1 was a marker of inflammatory activity and that higher serum HMGB1 levels were associated with destructive JIA and could be used as a marker of poor prognosis at the onset of disease.^142^ The JIA animal model revealed that in addition to high levels of expression, HMGB1 also exhibits nucleocytoplasmic translocation and extracellular release as the disease progresses,^143^ and analysis of human‐derived synovial fluid HMGB1 proved that active release of HMGB1 included acetylation‐dependent/independent mechanisms and various redox modifications, suggesting that HMGB1 plays an important role in the induction and maintenance of inflammatory events in the destructive process of chronic arthritis.^144^

#### 5.5.2 Systemic lupus erythematosus

Similar to those in JIA, there were dramatic changes in the upregulation of serum HMGB1 and the downregulation of sRAGE in children with systemic lupus erythematosus (SLE).^140^ Serum HMGB1 was positively correlated with IFN‐α, both of which were positively correlated with SLEDAI and ECLAM scores.^145^ The serum HMGB1 levels of children with lupus nephritis were significantly higher than those of normal renal patients. HMGB1 was positively correlated with the SLE activity index and 24‐h urine protein level but was negatively correlated with the creatinine clearance rate.^146^ When HMGB1 ≥ 40 g/L, serum HMGB1 showed good diagnostic value for SLE.^146^ Garcia‐Romo et al.,^147^ believed that the participation of HMGB1 in the pathogenesis of SLE was partially related to the NETs produced in SLE because NETs contain DNA and a large amount of LL37, HMGB1, and neutrophil proteins. These proteins promote the uptake and recognition of mammalian DNA by plasma cell‐like DCs, and activated pDCs participate in the pathological process of SLE by producing high levels of IFN‐α in a DNA‐ and TLR9‐dependent manner.

#### 5.5.3 Kawasaki disease

Kawasaki disease (KD) is an acute febrile, self‐limiting vasculitis in children, with coronary artery lesions (CALs) associated with 15%–25% of untreated patients.^148^ KD symptoms are related to the overactivation of the immune system caused by infection in children with genetic susceptibility.^149^ The serum HMGB1 levels in children with KD were significantly higher than those in healthy controls, reached a peak in the early acute phase, and then gradually declined with decreasing body temperature; moreover, the gene level of the reciprocal receptor RAGE also increased significantly.^150^ HMGB1 levels also reflected the responsiveness of children with KD to intravenous immunoglobulin (IVIG) therapy, which was based on the reports of Eguchi et al.^151^ showing that children with poor responses to high‐dose IVIG had significantly higher levels of HMGB1 than those with effective treatment. Ahn et al.^152^ further confirmed in their latest study that rs1412125 in HMGB1 may be a risk factor for CAL and IVIG resistance in patients with KD. By measuring the level of HMGB1 and its genetic polymorphism, it may be possible to provide potential markers to reveal IVIG resistance in advance.

#### 5.5.4 Macrophage activation syndrome

Macrophage activation syndrome (MAS) can be induced by systemic immune system diseases described above, especially the systemic type of JIA.^153^, ^154^ In a study of four children with severe MAS, tandem mass spectrometry analysis showed significant increases in ds‐HMGB1 isoform levels in the early stages of MAS.^155^ When HMGB1 levels fell sharply and indicated conversion to the os‐HMGB1 isoform, disease control coincided with supplementary etoposide therapy initiated to boost apoptotic cell death; when systemic HMGB1 levels drastically declined and the molecule was mainly present in its oxidized, noninflammatory isoform, systemic IFN‐γ, and ferritin peaked concomitantly with HMGB1.^155^ These observations encourage further studies of ds‐HMGB1 antagonists to improve the outcome of MAS.

#### 5.5.5 Henoch–Schönlein purpura

Henoch–Schönlein purpura (HSP) is now known as IgA vasculitis, which is a kind of leukocytoclastic vasculitis involving small vessels and primarily affects children, with an annual incidence of 13–20 cases per 100,000 children less than 17 years old.^156^ HSP is usually self‐limited, but some may cases be complicated by severe nephritis. Sporadic studies on HMGB1 in HSP showed that HMGB1 was significantly increased in the serum of children with HSP and abundantly expressed in the cytoplasm of endothelial cells in the damaged skin of HSP children.^157^ In addition, in vitro experiments showed that HMGB1 induced the release of TNF‐α and IL‐6 in human dermal microvascular endothelial cell lines.^157^ Wang et al.^158^ further showed that HMGB1 in children with HSP complicated by renal damage as higher than that in children with normal kidneys. HMGB1 was hypothesized to participate in the pathogenesis of HSP and HSP nephritis in children by inducing endothelial inflammation.

#### 5.5.6 Allergic rhinitis

Allergic rhinitis (AR) is a persistent IgE‐mediated inflammation of the nasal mucosa caused by allergen exposure that is characterized by an inflammatory response, and proinflammatory mediators play important roles in the progression of nasal inflammation.^159^ The level of HMGB1 in nasal lavage fluid of AR children was higher than that of healthy peers, and there was a strong correlation between the HMGB1 level and visual simulation score, which was further found to be more obvious in the severe symptom group through subgroup analysis.^160^ The animal model of AR was also used to show that after the inhibition of HMGB1, the expression and release of HMGB1 were decreased, and the changes in airway eosinophils, TH‐2 cytokines, total IgE, and goblet cell proliferation were also suppressed, together with improvements in AR symptoms. These results indicate that HMGB1 is a potential therapeutic target for AR.

#### 5.5.7 Vernal keratoconjunctivitis

Vernal keratoconjunctivitis (VKC) is a serious ocular inflammatory disease characterized by bilateral, chronic, and vision‐threatening effects and mainly affects children. VKC is highly seasonal, and severe and inappropriate treatment of VKC always leads to grievous ocular complications, such as glaucoma, corneal scarring, and blindness.^161^ Compared with that of healthy children, HMGB1 was highly expressed in the serum and lacrimal fluid of VKC patients,^162^, ^163^ and serum sRAGE was also increased. Effective therapy could reduce HMGB1 and sRAGE levels,^163^ proving that HMGB1 signaling plays a role in the pathogenesis of VKC.

### 5.6 HMGB1 and diseases of the endocrine system

There are only scattered reports of HMGB1 in pediatric endocrine system diseases. An important risk factor for metabolic syndrome (MS) in children is obesity, the incidence of which increases with the severity of obesity, and MS occurs in half of obese children.^164^ HMGB1 levels in obese children were significantly higher than those in the control group and were independently correlated with body mass index, IL‐23, IL‐6, free triiodothyronine, high‐density lipoprotein, and homeostasis model assessment of insulin resistance. ROC analysis showed that HMGB1 was more sensitive and specific than IL‐6 and adiponectin in identifying MS, indicating that HMGB1 may be an important diagnostic marker for obesity‐related complications (such as MS).^165^

### 5.7 HMGB1 and cancer

HMGB1 has complex functions in cancer, and it has considerable importance in tumor formation, progression, and the response to chemotherapy.^166^ HMGB1 is often overexpressed in the nuclei of cancer cells and embryonic cells,^167^ and this overexpression, along with dysfunction, is considered a marker of tumor and cancer progression.^168^ Autophagy has recently received increasing attention due to its role in conferring resistance to various commonly used anticancer therapies.^169^ HMGB1 and p53 form a complex to regulate the balance between cancer cell death and survival; autophagy induced by HMGB1 plays an important role in cancer.^170^

#### 5.7.1 Osteosarcoma

Osteosarcoma is the most common malignant bone tumor in children and adolescents and is characterized by high malignancy, easy early metastasis, and high lethality.^171^ The expression of HMGB1 in osteosarcoma tissues was significantly higher than that in normal bone tissues. Patients with lung metastases had higher HMGB1 levels than those without lung metastases.^172^ The inhibition of HMGB1 could inhibit the proliferation and invasion of human osteosarcoma MG‐63 cells and promote apoptosis of these cells in vitro.^172^ In chemotherapy for osteosarcoma, HMGB1 binds to the autophagy regulator Beclin1 and regulates the formation of the Beclin1–PI3KC3 complex, which induces autophagy to increase drug resistance.^173^ Certain noncoding RNAs (i.e., microRNA 22 [miRNA‐22])^174^ and drugs (i.e., 3,4‐dihydroxy‐9,10‐secoandrosta‐1,3,5,7‐tetraene‐9,17‐dione)^175^ enhance the sensitivity of osteosarcoma cells to chemotherapy by inhibiting HMGB1‐induced autophagy; therefore, HMGB1 plays an important role in the occurrence, progression, and sensitivity of osteosarcoma to chemotherapy.

#### 5.7.2 Retinoblastoma

Similar to osteosarcoma, retinoblastoma is the most common malignant intraocular tumor in infants and childhood, usually appearing in infants under 3 years of age, with high malignancy and metastasis and a high fatality rate.^176^, ^177^ HMGB1 is expressed in most retinoblastoma tissues, and its expression is significantly different in tissues with poor tumor differentiation and optic nerve infiltration.^178^ Since chemotherapeutic resistance often leads to treatment failure or a high recurrence rate, the mechanism of HMGB1 resistance in retinoblastoma has also been investigated. The overexpression of miRNA‐34A or direct interference in HMGB1 expression can significantly promote apoptosis by downregulating NF‐κB expression and inhibiting chemotherapeutic drug‐induced autophagy, thereby increasing the sensitivity of retinoblastoma to chemotherapy drugs.^179^, ^180^

#### 5.7.3 Neuroblastoma

Neuroblastoma is the most common extracranial tumor in childhood and the most common tumor in infants and young children; most cases occur before 2 years of age.^181^ Through analysis of the NCBI GEO database, HMGB1 overexpression was found in tumor tissues of up to 11% of children with neuroblastoma, who showed higher risk of tumor progression, recurrence, or death, possibly due to the immune escape triggered by tumor‐derived HMGB1 that is responsible for Treg phenotypic induction.^182^ In addition, HMGB1 inhibited PTEN by upregulating the miR‐221/222 cluster in neuroblastoma cell lines to induce a malignant phenotype.^183^ Autophagy induced by HMGB1 overexpression in Schwann cells also contributes to the proliferation of neuroblastoma cells,^184^ suggesting a role of HMGB1 in neuroblastoma and its potential as a therapeutic target.

#### 5.7.4 Leukemia

Serum HMGB1 levels are abnormally increased in the initial treatment stage of acute lymphoblastic leukemia and drop to normal levels in the complete remission stage after treatment.^185^ It is hypothesized that HMGB1 promotes the secretion of TNF‐α by leukemia cells through the activation of the MAPK signaling pathway and participates in tumor cell immune regulation.^185^ The abnormal expression of HMGB1 was positively correlated with the clinical status of childhood leukemia, which was attributed to the fact that endogenous HMGB1 induced autophagy and enhanced leukemia resistance through the phosphatidylinositol‐3‐kinase/protein kinase B/mammalian target of rapamycin complex 1 pathway.^186^ Similarly, HMGB1 was also significantly increased in children with acute promyelocytic leukemia (APL), but in the treatment of APL, differentiation therapy based on all‐trans retinoic acid (ATRA) and arsenic trioxide could induce excessive inflammation and differentiation syndrome (DS),^187^ which results in fatal effects. HMGB1 activated the MEK/ERK signal to promote the expression of inflammatory factors, and an HMGB1 neutralizing antibody inhibited the expression of ICAM‐1 in an ATRA‐treated DS mouse model and reduced mortality.^188^ Additionally, HMGB1 also induced acute myeloid leukemia cells to release inflammatory factors and activated the receptor Tim‐3 to induce the secretion of vascular endothelial growth factor, which proves that HMGB1 contributes to the survival/proliferation of AML cells and that angiogenesis ultimately promotes the progression of AML.^189^

#### 5.7.5 Non‐Hodgkin's lymphoma

The gene expression level of HMGB1 was upregulated in the lymph nodes of children with Non‐Hodgkin's lymphoma (NHL) compared with normal tissues, and an HMGB1‐positive reaction was only found in lymphoma cells.^190^ Although no correlation between HMGB1 expression and NHL grading was found, a high percentage of lymphomas exhibit HMGB1 overexpression, which can support the growth and angiogenesis of lymphoma cells in a paracrine way when released (e.g., due to necrosis).^190^

### 5.8 HMGB1 and skin diseases

#### 5.8.1 Erythema toxicum neonatorum

Although the pathogenesis of erythema toxicum neonatorum (ETN) has not been well elucidated, it is believed that this disease may be triggered by the allergic mechanism and the inflammatory response to microbial colonization,^191^ manifesting as a transient skin disease characterized by erythema, papules, and pustules. ETN pustules are dominated by infiltrating eosinophils.^192^ Marchini et al.^193^ found the nucleocytoplasmic relocation of HMGB1 in keratinocytes and macrophages, and HMGB1 was also found in melanocyte cytoplasm, indicating that the immune response mediated by the release of HMGB1 from the nucleus is involved in the occurrence of ETN.

#### 5.8.2 Atopic eczema/dermatitis syndrome

Atopic eczema/dermatitis syndrome (AEDS) is a chronic relapsing–relieving alternating inflammatory skin disease that usually begins in early childhood and is characterized by damage to the skin's barrier function leading to epidermal damage and changes in the permeability of the skin to allergens and microorganisms.^194^ According to whether it is related to IgE sensitization, AEDS can be divided into atopic AEDS (aAEDS) or nonatopic AEDS (naAEDS); a study showed that there were no differences in HMGB1 levels between children with aAEDS and naAEDS, but both were significantly higher than those of the healthy control group.^195^ Among children with aAEDS and naAEDS, those with severe score atopic dermatitis values had increased levels of HMGB1, indicating that HMGB1 was a biomarker of the severity of AEDS.^195^

#### 5.8.3 Epidermolysis bullosa

Epidermolysis bullosa (EB) is a group of hereditary skin diseases caused by a lack of protein or structural abnormalities at the skin‐epidermal junction. Insufficient protein causes the skin to become brittle and vulnerable to mechanical stimulation, producing blisters or erosions, and there is currently no specific therapy.^196^ In a study of EB in adults and children, it was found that serum CXCL12 and HMGB1 levels were significantly increased while CCL21 levels were decreased; moreover, immunofluorescence confirmed that CCL21, CCL27, HMGB1, and CXCL12 were more extensively expressed in the tissues of EB patients than in control tissues, indicating that fluctuations in chemokine levels may promote the wound healing process in a coordinated manner.^197^

### 5.9 HMGB1 and chemical/drug‐induced toxic damage

Some chemicals in the environment, such as heavy metals and various drugs, easily cause damage to children, especially to the nervous system and immune system, and can even cause tumors.^198^, ^199^ The inflammatory response plays an important role in this damage. It has been reported that lead,^200^ chlorpyrifos^201^ and isoflurane^202^ can cause damage to the nervous system in children. Animal or cellular models have shown that the abnormal increased expression of HMGB1 may play a role in the abnormal neuroinflammatory response triggered by these toxic substances.

### 5.10 HMGB1 and other reported diseases

In addition to the changes detected in the diseases summarized above, the role of HMGB1 in other diseases has been poorly reported. Circulating HMGB1 levels were found to be significantly elevated in children with sickle cell disease (SCD) and model animals, and the level was further increased in the occurrence of acute sickling events (vasoocclusive crises in humans or hypoxia/reoxygenation injury in mice), suggesting that HMGB1 plays a role in SCD‐mediated inflammation by activating TLR4.^203^ In children who require peritoneal dialysis, peritonitis is the most important complication associated with peritoneal dysfunction and treatment failure,^204^ and measuring new biomarkers of proinflammatory or anti‐inflammatory factors and fibrosis in dialysate can be used as a noninvasive method for the indirect evaluation of the peritoneum. The serum and peritoneal fluid HMGB1 levels peak in the early stage of acute peritonitis and gradually decrease with treatment, suggesting that HMGB1 may be used as a biomarker to evaluate peritoneal status and therapy response.^205^ Finally, alcohol abuse in adolescence leads to increased expression of HMGB1 and TLRs/RAGE in the prefrontal cortex in adulthood, and this activated neuroinflammatory response plays an important role in the mechanism of alcohol addiction.^206^, ^207^

## 6 CONCLUSIONS AND PERSPECTIVES

In pediatric diseases other than cancer, HMGB1 mainly acts as DAMP to trigger the inflammatory response, while in cancers, HMGB1 plays roles in promoting tumor proliferation and metastasis mainly by inducing autophagy and inhibiting apoptosis. In most diseases, the level of HMGB1 is increased, which in turn stimulates inflammation and autophagy. As the proinflammatory effect of HMGB1 is closely related to its release into the extracellular space, HMGB1 can be restricted to the nucleus by regulating its posttranscriptional modification to exert a therapeutic effect in the future. In addition, the signaling pathway of HMGB1 can be blocked to reduce its pathogenicity. The role of HMGB1 in pediatric diseases requires more in‐depth research, and the development of models that are more consistent with the characteristics of these diseases is needed to explore the specific molecular mechanisms of the involvement of HMGB1 in these diseases.

## CONFLICT OF INTERESTS

The authors declare that there are no conflict of interests.

## AUTHOR CONTRIBUTIONS

Kai Le and Bo Li contributed to conception and design of the study and drafted the article. Kai Le, Bo Li, Xin Peng, He Li and Fei Chen contributed to search the database and extract the required information. Yuxia Chen and Yingqian Zhang edited and revised the whole manuscript. All authors read and approved the final version of the manuscript.
